# Effects of Short-Chain Fatty Acid Modulation on Potentially Diarrhea-Causing Pathogens in Yaks Through Metagenomic Sequencing

**DOI:** 10.3389/fcimb.2022.805481

**Published:** 2022-03-23

**Authors:** Kun Li, Zhibo Zeng, Juanjuan Liu, Lulu Pei, Yaping Wang, Aoyun Li, Muhammad Fakhar-e-Alam Kulyar, Muhammad Shahzad, Khalid Mehmood, Jiakui Li, Desheng Qi

**Affiliations:** ^1^ College of Veterinary Medicine, Huazhong Agricultural University, Wuhan, China; ^2^ Institute of Traditional Chinese Veterinary Medicine, College of Veterinary Medicine, Nanjing Agricultural University, Nanjing, China; ^3^ MOE Joint International Research Laboratory of Animal Health and Food Safety, College of Veterinary Medicine, Nanjing Agricultural University, Nanjing, China; ^4^ Department of Animal Nutrition and Feed Science, College of Animal Science and Technology, Huazhong Agricultural University, Wuhan, China; ^5^ Faculty of Veterinary and Animal Sciences, The Islamia University of Bahawalpur, Bahawalpur, Pakistan

**Keywords:** yak, diarrhea, gut microbiota, metagenomics, SCFA

## Abstract

Short-chain fatty acids (SCFA) are principal nutrient substrates of intestinal epithelial cells that regulate the epithelial barrier in yaks. Until now, metagenomics sequencing has not been reported in diarrheal yaks. Scarce information is available regarding the levels of fecal SCFA and diarrhea in yaks. So, our study aims to identify the potential pathogens that cause the emerging diarrhea and explore the potential relationship of short-chain fatty acids in this issue. We estimated diarrhea rate in yaks after collecting an equal number of fecal samples from affected animals. Metagenomics sequencing and quantitative analysis of SCFA were performed, which revealed 15%–25% and 5%–10% prevalence of diarrhea in yak’s calves and adults, respectively. Violin box plot also showed a higher degree of dispersion in gene abundance distribution of diarrheal yaks, as compared to normal yaks. We found 366,163 significant differential abundance genes in diarrheal yaks, with 141,305 upregulated and 224,858 downregulated genes compared with normal yaks *via* DESeq analysis. Metagenomics binning analysis indicated the higher significance of bin 33 (Bacteroidales) (*p* < 0.05) in diarrheal animals, while bin 10 (*p* < 0.0001), bin 30 (Clostridiales) (*p* < 0.05), bin 51 (Lactobacillales) (*p* < 0.05), bin 8 (Lachnospiraceae) (*p* < 0.05), and bin 47 (Bacteria) (*p* < 0.05) were significantly higher in normal yaks. At different levels, a significant difference in phylum (*n* = 4), class (*n* = 8), oder (*n* = 8), family (*n* = 16), genus (*n* = 17), and species (*n* = 30) was noticed, respectively. Compared with healthy yaks, acetic acid (*p* < 0.01), propionic acid (*p* < 0.01), butyric acid (*p* < 0.01), isobutyric acid (*p* < 0.01), isovaleric acid (*p* < 0.05), and caproic acid (*p* < 0.01) were all observed significantly at a lower rate in diarrheal yaks. In conclusion, besides the increased *Staphylococcus aureus*, *Babesia ovata*, *Anaplasma phagocytophilum*, *Bacteroides fluxus*, viruses, *Klebsiella pneumonia*, and inflammation-related bacteria, the decrease of SCFA caused by the imbalance of intestinal microbiota was potentially observed in diarrheal yaks.

## Introduction

The long-haired bovine species, i.e., the yak, is an indispensable economic pillar on the Qing-Tibetan plateau (Li et al., 2014). There are approximately 15 million yaks in China, accounting for over 90% of the world’s yak population (Li et al., 2018; Li et al., 2020). At an average of 3,000–5,000 m above sea level, the yak is depicted as a symbolic animal dependent on herdsmen’s lives (Li et al., 2014). Yaks serve as transportations, especially in the mountainy zigzag areas (Li et al., 2017). Meat, butter, and milk from yaks are also considered essential food items for local Tibetans (Li et al., 2018). Its hide is used to make boots, rafts, aprons, leather bags, and leather harnesses (Li et al., 2020). While the long hairs and dungs are commonly used for livelihood purposes (Li et al., 2017).

Hongyuan is located on the eastern edge of the Qinghai-Tibetan plateau and northwest of Sichuan province, with a northern latitude of 31°51′–33°33′ and eastern longitude of 101°51′–103°22′. In this continental plateau, the temperature ranges from −22.8°C to 24.6°C, with an average temperature of 2.9°C and 860.8 mm precipitation annually. According to the latest reports, animal husbandry is the primary industry with 75,383 yak population in this region. About 7,765 kg of yak’s meat has also been produced annually, accounting for 96.23% of the total meat production in Hongyuan (Bureau of Statistics of Hongyuan County, http://www.hongyuan.gov.cn/hyxrmzf/c100057/zwgk.shtml). However, an emerging endemic diarrheal disease in yaks during the past few years (usually from May to August) has caused deaths to animals and caused a huge constraint on the development of the local economy.

Bovine diarrhea is a common disease throughout farms with worldwide distribution. It has been causing a heavy economic loss concerning fertility rate, milk production, and animal growth (Han et al., 2017). In yaks, diarrhea-causing pathogens, i.e., *Cryptosporidium parvum*, bovine viral diarrhea (BVD) virus, and *Escherichia coli* have been reported previously (Deng et al., 2015; Qi et al., 2015; Li et al., 2018). Although many measures have been employed to improve hygiene and feeding management with extensive drugs, the problem is still at its peak (Cho et al., 2013).

Intestinal tract is colonized by a large and diverse type of microbial microbiota (Xu et al., 2019). This community produces extensive metabolic products in the intestine, which interact intimately with host cells to maintain physiological processes and functions, i.e., nutrition absorption, host metabolism, and immunity (Backhed et al., 2012; Xu et al., 2019; Wei et al., 2020). Mainly, microbiota benefits the host through intestinal epithelium by protecting it and producing beneficial metabolites, which helps in food digestion and against pathogenic invasion. The gut microbiome can convert fermentable dietary fibers into short-chain fatty acids (SCFA) that provide additional energy to the host (Blaut, 2015). These SCFA are organic carboxylic acids with less than 6 carbon atoms of the acetate; propionate and butyrate are the most abundant extension in the intestine (Alam et al., 2000). A previous study reported that diets containing alfalfa meal and commodity-concentrated fiber could drop diarrhea rate *via* metabolic interactions between hindgut microbiota and SCFA in piglets (Liu et al., 2018). These SCFA act as ligands for G-protein coupled receptors by activating anti-inflammatory signaling (Parada Venegas et al., 2019). Metagenomics sequencing is commonly utilized in microbial organisms, as it provides the accurate classification of microbiota species and annotation to the bacteria at a functional level rather than functional prediction (Mukhuba et al., 2019; Luan et al., 2020; Tian et al., 2020).

It is generally accepted that microbiota composition and function contribute to the host’s health status (Long et al., 2020). Previously, dysfunctional gut microbiota was related to diseases like human inflammatory bowel disease, diabetes, and cardiovascular disease (Wang et al., 2011; Wei et al., 2020). The imbalance of such intestinal microbiota may cause diarrhea due to the growing conditional pathogens, mucosal barrier damage, immunity dropping, and intestinal permeability (Long et al., 2020). However, it is still unclear how the changed microbiota can cause the emerging diarrheal disease in yaks. Hence, this study was conducted to explore such potential pathogens and short-chain fatty acid changes in diarrheal yaks.

## Materials and Methods

### Sample Collection

We visited 10 family yak farms with diarrhea outbreaks during June and July 2019 (**Table 1**) in Hongyuan, Sichuan, China. The prevalence of diarrhea in farms was estimated by consulting animal owners as these bovines were free ranged, having grasslands without concentrated feed on the plateau. A total of 100 fresh fecal samples were collected from diarrheal (*n* = 60) and healthy (*n* = 40) yak calves in 2~3 months. All the fecal samples were frozen immediately in liquid nitrogen and then transported to the laboratory of Huazhong Agricultural University, Wuhan, China. Samples were kept at −80°C for further analysis.

**Table 1:** Estimation of the prevalence of diarrhea in yaks in Hongyuan on the plateau.

Farms	No. of yak calves (prevalence %)	No. of adult yaks (prevalence %)	Total No. of yak (prevalence %)
1	20–25 (10%–20%)	40–50 (2%–8%)	60–75 (5%–20%)
2	80–100 (25%–30%)	220–250 (5%–18%)	300–350 (10%–20%)
3	30–40 (5%–8%)	20–25 (0)	50–65 (2%–10%)
4	25–30 (2%–4%)	40–50 (0)	65–80 (0%–4%)
5	150–200 (15%–20%)	200–220 (5%–10%)	350–420 (10%–15%)
6	30–50 (10%–15%)	30–40 (2%–5%)	60–90 (5%–10%)
7	20–30 (5%–10%)	20–25 (2%–4%)	40–55 (4%–10%)
8	20–35 (10%–15%)	30–35 (5%–10%)	50–70 (8%–12%)
9	60–70 (20%–30%)	50–60 (5%–10%)	110–130 (15%–20%)
10	20–25 (10%–15%)	30–40 (3%–5%)	50–65 (8%–10%)
Total	455–605 (15%–25%)	680–795 (5%–10%)	1,135–1,400 (10%–15%)

### DNA Extraction and Mixing of Samples

The genomic DNA from fecal samples were extracted by QIAamp Fast DNA Stool Mini Kit (QIAGEN, Venlo, NL) following the manufacturer’s instructions. Genomic DNA samples were stored at −20°C before further assessment. The quantity and quality of extractions were measured using a NanoDrop ND-1000 spectrophotometer (Thermo Fisher Scientific, Waltham, MA, USA), agarose gel electrophoresis, and Quant-iT PicoGreen dsDNA Assay Kit (Invitrogen). Every 10 samples from different farms were then mixed and gained 6 diarrheal group samples (D1, D2, D3, D4, D5, D6) and 4 normal group samples (NA, NB, NC, ND).

### Library Construction and Sequencing

Metagenome shotgun sequencing libraries (400 bp) were constructed using Illumina TruSeq Nano DNA LT Library Preparation Kit. Each library was sequenced by employing the Illumina HiSeq X-ten platform (Illumina, USA) with PE150 strategy (Shanghai, China).

### Sequence Analysis

Further analysis to achieve quality-filtered reads, the sequencing adapters were removed from raw sequencing reads by using Cutadapt (v1.2.1) (Martin, 2011). Low-quality reads were trimmed by performing a sliding window algorithm. Reads were aligned to the host genome *via* BWA (http://bio-bwa.sourceforge.net/) to remove host gene contamination (Li and Durbin, 2009). Quality-filtered reads were then *de novo* assembled to construct the metagenome for each mixed sample based on the iterative De Bruijn graph assembler for sequencing data with highly uneven depth (IDBA-UD) (Peng et al., 2012). All coding regions (CDS) of metagenomic scaffolds longer than 300 bp were predicted by MetaGeneMark (http://exon.gatech.edu/GeneMark/metagenome) (Zhu et al., 2010). CDS sequences samples were clustered by CD-HIT at 90% protein sequence identity to obtain a nonredundant gene catalog (Fu et al., 2012). Gene abundance in each sample was estimated (http://soap.genomics.org.cn/) based on aligned read number. The lowest common ancestor taxonomy of the nonredundant genes was obtained by aligning them against the NCBI-NT database by BLASTN (*p*-value <0.001). Similarly, the functional profiles of the nonredundant genes were obtained and annotated in public databases including the Golang (GO), Kyoto Encyclopedia of Genes and Genomes (KEGG), Evolutionary genealogy of genes were done through: Non-supervised Orthologous Groups (EggNOG), and Carbohydrate-Active enZYme (CAZy) by utilizing DIAMOND (Buchfink) alignment algorithm (Buchfink et al., 2015). The normalization of reads per sample was performed prior to statistical analysis to ensure that bias is not caused by sampling at different depths.

### Comparing the Difference of Intestinal Microbiota Between Normal and Diarrheal Yaks

Based on the taxonomic and functional profiles of nonredundant genes, linear discriminant analysis effect size (LEfSe) was performed to detect differentially abundant taxa and functions across the groups using default parameters (Segata et al., 2011). Beta diversity analysis was performed to investigate microbial communities’ compositional and functional variations across diarrheal and healthy yak samples through Bray–Curtis distance metrics (Bray and Curtis, 1957). Visualization was done *via* principal coordinate analysis (PCoA), nonmetric multidimensional scaling (NMDS), and unweighted pair-group method with arithmetic mean (UPGMA) hierarchical clustering (Ramette, 2007). Differential gene (upregulated gene and downregulated gene) abundance analysis was performed *via* DESeq at fold change ≥2 and *p*-value <0.01 (Backhed et al., 2015). Metagenomics binning analysis was carried out using Maxbin2 and Maxbat2 (Sieber et al., 2018) at >50% genome completeness and <10% contamination rate.

### Extraction of Fatty Acids From Fecal Samples

Firstly, 20 mg from each sample was taken out and combined with 1 ml phosphoric acid (0.5% *v*/*v*) in a sterile 2-ml EP tube and then mixed thoroughly *via* vortex and ultrasonication for 10 and 5 min, respectively. Secondly, a 0.1-ml sample was taken out and then placed in a sterile 1.5-ml EP tube along with 0.5 ml MTBE (CAS No. 1634-04-4). The final product was mixed thoroughly *via* vortex and ultrasonication for 3 and 5 min, respectively. Thirdly, at 12,000 rpm, the sample was centrifuged at 4°C for 10 min. After taking out 0.2 ml from the supernatant, 10 extracted samples from the same group were mixed and vortexed for 1 min. Finally, a 0.2-ml mixture was taken out and transferred into a vial for sample detection and further analysis using GC-MS/MS (Agilent).

### Qualitative and Quantitative Analysis of SCFA

Total ion current (TIC) and standard quality of all mixture samples were detected through GC-MS/MS (Agilent) using the procedures and parameters shown in **Table 2**. Quality control analysis of the samples was carried out to ensure the method’s validity. The standard quality of the samples was checked thrice to measure the instrument stability. This standard quality was tested in every ten samples to monitor the repeatability of the analysis process. Qualitative and quantitative analyses of SCFA were performed by Agilent MassHunter. Standard curves for all SCFA were generated by detecting standard quality control samples, which were caproic acid (CAS No. 64-19-7), isovaleric acid (CAS No. 79-09-4), valeric acid (CAS No. 79-31-2), butyric acid (CAS No. 107-92-6), propionic acid (CAS No. 503-74-2), acetic acid (CAS No. 109-52-4), isobutyric acid (CAS No. 142-62-1), 2-methylpentanoic acid (CAS No. 97-61-0), MTBE (CAS No. 1634-04-4), and phosphoric acid (CAS No. 7664-38-2).

**Table 2:** Parameters employed in GC-MS/MS.

Procedure	Parameter
Sample load	2 µl
Front inlet mode	Splitless
Carrier gas	Helium
Column	DB-FFAP (30 m × 0.25 mm × 0.25 μm)
Column flow	1.2 min^−1^
Oven temperature ramp	95°C hold on 1 min, raised to 100°C at a rate of 25°C/min, raised to 130°C at a rate of 17°C/min, hold on 0.4 min, raised to 200°C at a rate of 25°C/min, hold on 0.5 min, after running for 3 min
Front injection temperature	200°C
Transfer line temperature	230°C
Ion source temperature	230°C
Quad temperature	150°C

### Statistical Analysis

The prevalence of diarrhea in different farms was analyzed through IBM SPSS Statistics (SPSS 22.0) using Chi-square (results were followed up as upper and lower limits of prevalence). Quantitative analyses of SCFA were expressed as means ± standard deviation (SD). Whereas, the difference of SCFA among the groups was analyzed *via* Wilcoxon test and fold changes through piloting SPSS (IBM, 22.0). *p*-Values <0.05 were considered statistically significant. *t*-Test was also performed to compare intestinal microbiota differences using IBM SPSS Statistics. Annotated analysis was performed *via* MetaPhlAn2 (http://huttenhower.sph.harvard.edu/metaphlan2, Version 2.0) compared with the database (Kanehisa et al., 2014). Functional profile analysis was performed by annotating against the GO, KEGG, EggNOG, and CAZy databases (Kanehisa et al., 2006; Cantarel et al., 2009; Kanehisa et al., 2014; Powell et al., 2014).

## Results

### Data Deposition

The raw sequences data were deposited in the BioSample database with the accession number: SAMN16091789-SAMN16091798.

### Prevalence of Diarrhea in Yaks

Diarrhea in yaks was found in all farms (10/10), especially in calf farms (100%), while 80% (8/10) of farms reported diarrhea in adult yaks. The overall prevalence of diarrhea ranged from 0% to 4% and 15% to 20%. While in calves and adults, the prevalence ranged from 15% to 25% and 5% to 10%, respectively (**Table 1**). A significant difference was observed in both upper (*p* < 0.001) and lower limits of prevalence (*p* < 0.01) (**Supplementary Figure S2**).

### Sequencing Data of Yak Microbiota Samples and Gene Abundance Distribution

Overall, 445,199,120 total reads and 445,089,080 clean reads were obtained from diarrheal yaks, while 285,976,660 and 285,951,940 total and clean reads were obtained from normal yaks. Moreover, 66.30 and 42.67 Gb clean bases were found in diarrheal and normal yak groups, respectively. The Q20 and Q30 in both groups was more than 97% and 92%, which confirmed reliable and accurate base recognitions (Bolger et al., 2014). No significant difference (*p* > 0.05) was observed in total reads, clean reads, Q20, and Q30. However, a significant difference was found in GC content between diarrheal (44.69%~46.08%) and normal yaks (46.12%~46.38%) (*p* < 0.05) (**Supplementary Figure S3**). According to the violin box plot, the degree of dispersion in gene abundance distribution was higher in diarrheal than normal yaks (**Supplementary Figure S4**).

### Species Composition and Abundance Analysis

The abundance of Firmicutes and Proteobacteria was found to be significantly lower in yaks with diarrhea than in normal yaks (*p* < 0.05) (**Supplementary Figure S5**).

**Figure 1:**
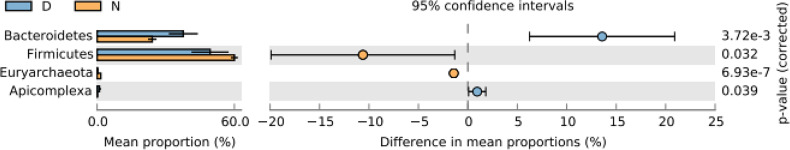
Comparing intestinal microbiota difference between different yaks at phylum level. D, diarrheal group samples; N, normal group samples.

Firmicutes and Bacteroidales were found primarily in both groups (**Supplementary Figure S6**). Principal component analysis (PCA) found the left-side location of D1, D2, D3, D4, D5, and D6 groups. While NA, NB, NC, and ND groups were located on the right side in two-dimensional graphic representation. Samples in normal yaks were concentrically distributed as compared with the diarrheal yaks (**Supplementary Figure S7A**). Compared with normal animals, Bacteroidetes (*p* < 0.01) and Apicomplexa (*p* < 0.05) were significantly higher in diarrheal yaks, while Firmicutes (*p* < 0.05) and Euryarchaeota (*p* < 0.001) were significant at lower levels (**Figure 1**).

Clostridia and Bacteroidia were observed mainly in normal yaks at the class level, while Bacteroidia, Clostridia, and Bacilli were dominated in diarrheal animals (**Supplementary Figure S8**). PCA showed that diarrheal and normal yak samples were infrequent and together, respectively (**Supplementary Figure S7B**). Compared with normal animals, *Aconoidasida* (*p* < 0.05) was significantly higher in diarrheal yaks, while *Clostridia* (*p* < 0.05), *Methanobacteria* (*p* < 0.001), *Flavobacteriia* (*p* < 0.001), *Deltaproteobacteria* (*p* < 0.001), *Alphaprotebacteria* (*p* < 0.01), and *Cytophagia* (*p* < 0.001) were significantly lower in number (**Supplementary Figure S9**).

At the order level, Clostridiales was higher in normal yaks, while Bacteroidales was higher in diarrheal animals (**Supplementary Figure S10**). PCA showed that the samples in diarrheal animals were far from each other than normal animals (**Supplementary Figure S7C**). In comparison with normal animals, *Bacteroidales* (*p* < 0.01) and *Piroplasmida* (*p* < 0.05) were significantly higher, while *Clostridiales* (*p* < 0.05), M*ethanobacteriales* (*p* < 0.001), *Flavobacteriales* (*p* < 0.001), *Cytophagales* (*p* < 0.001), *Spirochaetales* (*p* < 0.001), and *Marinilabiliales* (*p* < 0.001) were significantly lower in diarrheal yaks (**Supplementary Figure S11**).

At the family level, *Ruminococcaceae* and *Lachnospiraceae* were found mainly in normal yaks, while Bacteroidaceae was found in diarrheal yaks with high abundance (**Supplementary Figure S12**). PCA showed that samples in diarrheal animals were located far from each other than normal animals (**Supplementary Figure S7D**). Compared with normal animals, *Bacteroidaceae* (*p* < 0.001), *Staphylococcaceae* (*p* < 0.05), and *Babesiida*e (*p* < 0.05) were higher in diarrheal yaks, while *Ruminococcaceae* (*p* < 0.05), *Rikenellaceae* (*p* < 0.001), *Clostridiaceae* (*p* < 0.05), *Eubacteriaceae* (*p* < 0.001), *Methanobacteriaceae* (*p* < 0.001), *Oscillospiraceae* (*p* < 0.001), *Paenibacillaceae* (*p* < 0.001), *Flavobacteriaceae* (*p* < 0.001), *Muribaculaceae* (*p* < 0.001), *Spirochaetaceae* (*p* < 0.001), *Clostridiales* Family XIII. Incertae Sedis (*p* < 0.001), *Erysipelotrichaceae* (*p* < 0.05), and *Eggerthellacea*e (*p* < 0.001) were remarkably lower (**Supplementary Figure S13**).

**Figure 2:**
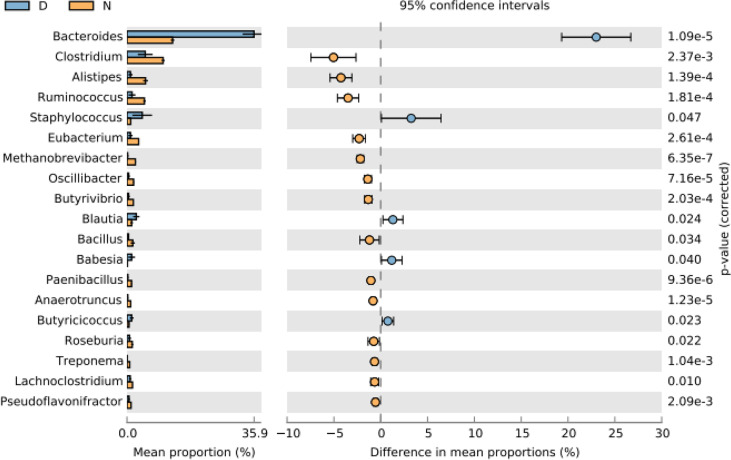
Comparing intestinal microbiota difference between different yaks at genus level. D, diarrheal group samples; N, normal group samples.

*Bacteroides* and *Clostridium* were found higher at the genus level in normal yaks, while *Bacteroides* was the main genus in diarrheal yaks (**Supplementary Figure S14**). PCA indicated that diarrheal samples were located far from normal animals (**Supplementary Figure S7E**). Compared with normal animals, *Bacteroides* (*p* < 0.001), *Staphylococcus* (*p* < 0.05), *Blautia* (*p* < 0.05), *Babesia* (*p* < 0.05), and *Butyricicossus* (*p* < 0.05) were outstandingly higher in diarrheal yaks, while *Clostridium* (*p* < 0.01), *Alistipes* (*p* < 0.001), *Ruminococcus* (*p* < 0.001), *Eubacterium* (*p* < 0.001), *Methanobrevibacter* (*p* < 0.001), *Oscilllibacter* (*p* < 0.001), *Butyrivibrio* (*p* < 0.001), *Bacillus* (*p* < 0.05), *Paenibacillus* (*p* < 0.001), *Anaerotruncu* (*p* < 0.001), *Roseburia* (*p* < 0.05), *Treponema* (*p* < 0.01), and *Lachnoclostridium* (*p* < 0.05) were lower (**Figure 2**).

**Figure 3:**
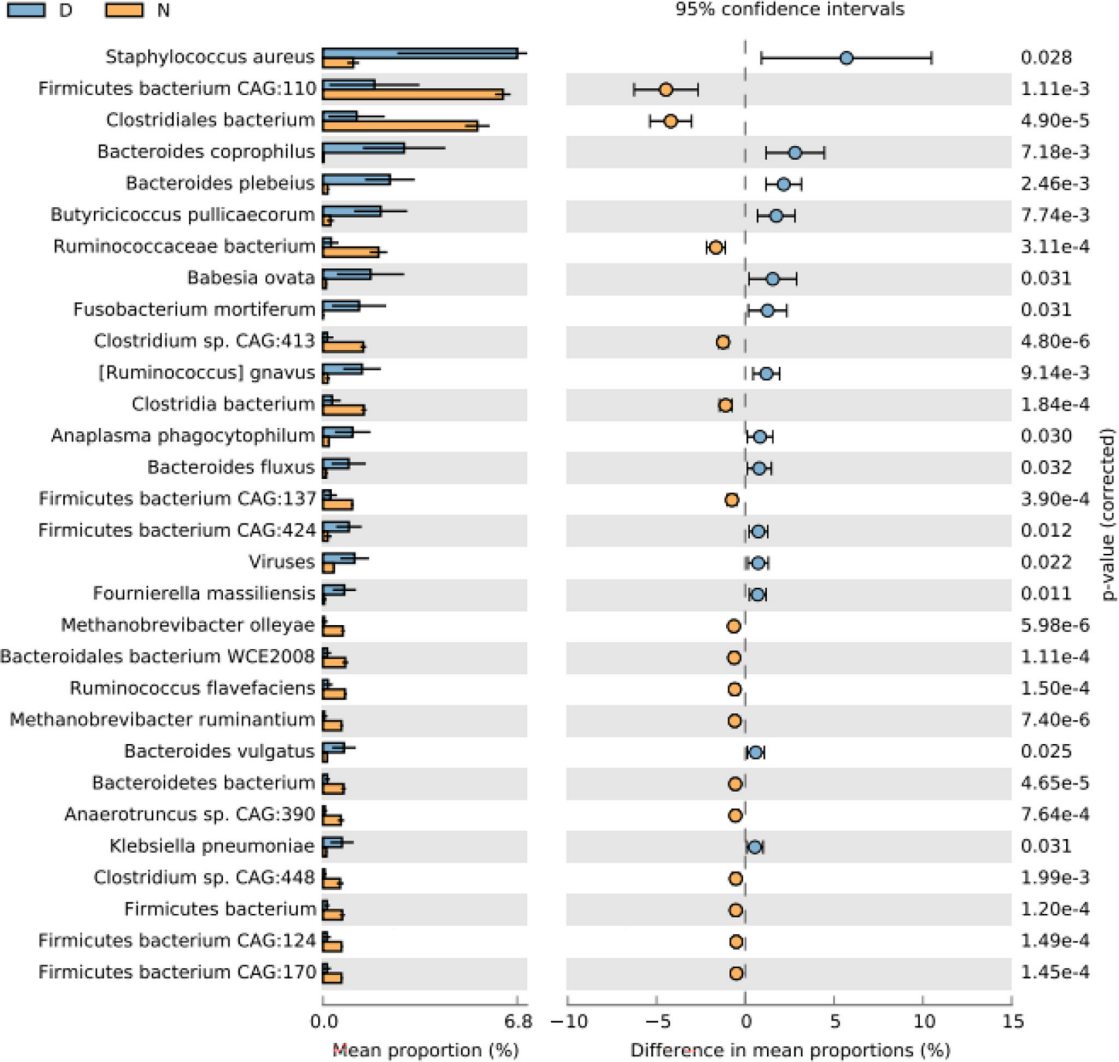
Comparing intestinal microbiota difference between different yaks at species level. D, diarrheal group samples; N, normal group samples.

At the species level, *Firmicutes bacterium* CAG:110 was found to be most abundant in normal yaks, while *Staphylococcus aureus* was the main species in diarrheal yaks (**Supplementary Figure S15**). PCA indicated that samples in diarrheal animals located separately as compared with normal animals (**Supplementary Figure S7F**). Compared with normal animals, *Staphylococcus aureus* (*p* < 0.05), *Bacteroides coprophilus* (*p* < 0.01), *Bacteroides plebeius* (*p* < 0.01), *Butyricicoccus pullicaecorum* (*p* < 0.01), *Babesia ovata* (*p* < 0.05), *Fusobacterium mortiferum* (*p* < 0.05), [*Ruminococcus*] *gnavus* (*p* < 0.01), *Anaplasma phagocytophilum* (*p* < 0.05), *Bacteroides fluxus* (*p* < 0.05), *Firmicutes bacterium* CAG:424 (*p* < 0.05), viruses (*p* < 0.05), *Fournierella massiliensis* (*p* < 0.05), *Bacteroides vulgatus* (*p* < 0.05), and *Klebsiella pneumoniae* (*p* < 0.05) were higher in diarrheal yaks, while *Firmicutes bacterium* CAG:110 (*p* < 0.01), *Clostridiales bacterium* (*p* < 0.001), *Ruminococcaceae bacterium* (*p* < 0.001), *Clostridium* sp. CAG:413 (*p* < 0.001), *Clostridia bacterium* (*p* < 0.001), *Firmicutes bacterium* CAG:137 (*p* < 0.001), *Methanobrevibacter olleyae* (*p* < 0.001), *Bacteroidales bacterium* WCE2008 (*p* < 0.001), *Ruminococcus flavefaciens* (*p* < 0.001), *Methanobrevibacter ruminantium* (*p* < 0.001), *Bacteroidete bacterium* (*p* < 0.001), *Anaerotruncus* sp. Cag:390 (*p* < 0.001), *Clostridium* sp. CAG:448 (*p* < 0.001), *Firmicutes bacterium* (*p* < 0.01), *Firmicutes bacterium* CAG:124 (*p* < 0.001), and *Firmicutes bacterium* CAG:170 (*p* < 0.001) were significantly lower in number (**Figure 3**).

**Figure 4:**
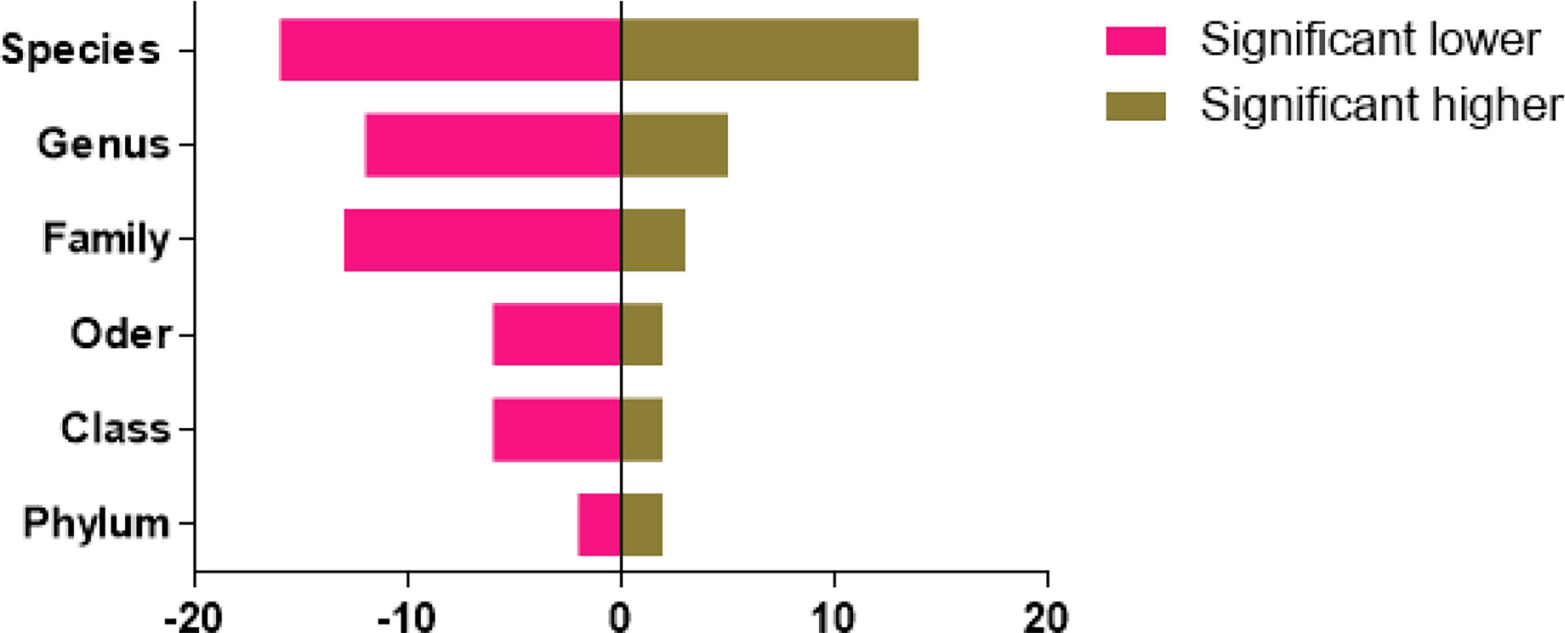
Statistics of significant species composition of diarrheal animals in different levels compared with normal animals (*x*-axis: difference numbers; *y*-axis: different levels).

**Figure 5:**
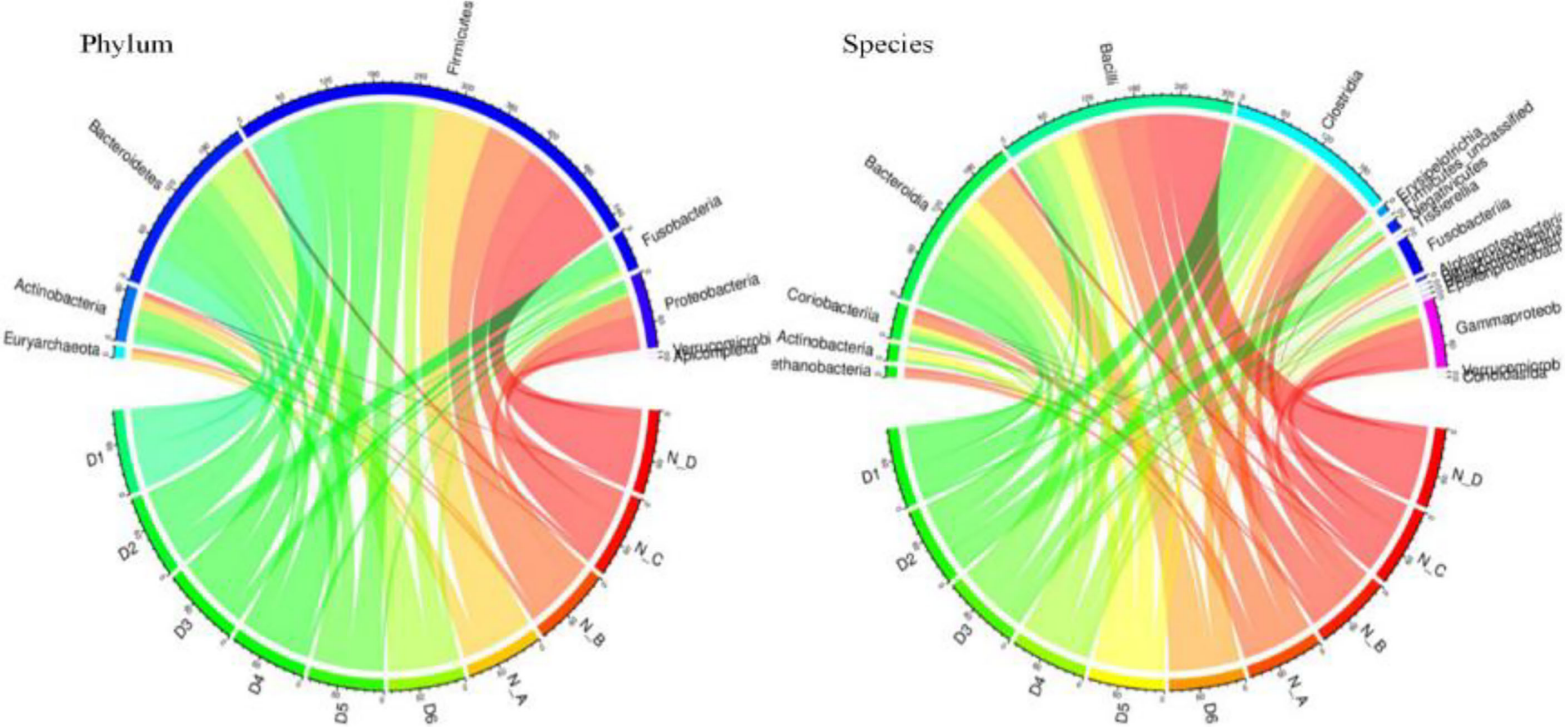
The relationship map of circus related to yaks' different species.

The statistics of significant compositional species compared with normal animals are shown in **Figure 4**. The circus map indicated that the phylum level of the two groups is mainly consisted of Firmicutes, while a significant difference was found in the case of Fusobacteria, Bacteroidetes, and Proteobacteria in both groups. While no significant difference was found in Bacteroidia, Bacilli, and Gammaproteo at the species level (**Figure 5**).

### Functional Analysis of Intestinal Yak Microbiota

**Figure 6:**
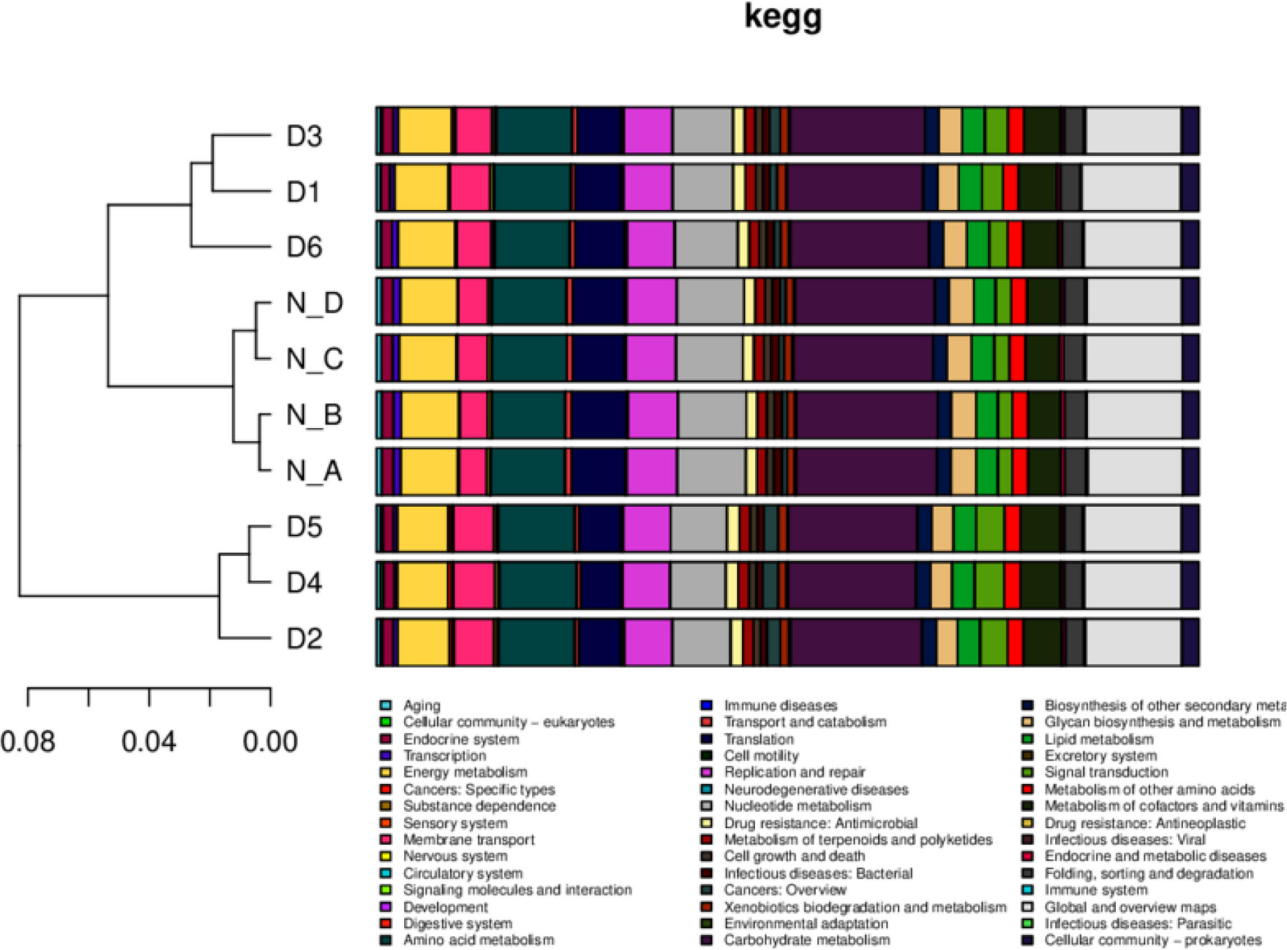
The relative functional abundance analysis. KEGG. D1, D2, D3, D4, D5, and D6 represent the diarrheal samples; N_A, N_B, N_C, and N_D represent the normal samples.

Annotated scores of one having HSP >60 bits were selected for analyzing relative abundance at different functional levels (Qin et al., 2012; Karlsson et al., 2012; Karlsson et al., 2013; Backhed et al., 2015). In total, 354,990, 486,219, 778,943, 867,820, 366,984, and 188,719 nonredundant genes were found in the GO, eggnog, KEGG, NR, Swissport, and CAZy databases, respectively. In KEGG, nonredundant genes were related to cellular community and energy metabolism and 40 more metabolic pathways in all yaks. Nonsignificantly, lower nervous system, development, and nucleotide metabolism were found in diarrheal yaks (**Figure 6**).

**Figure 7:**
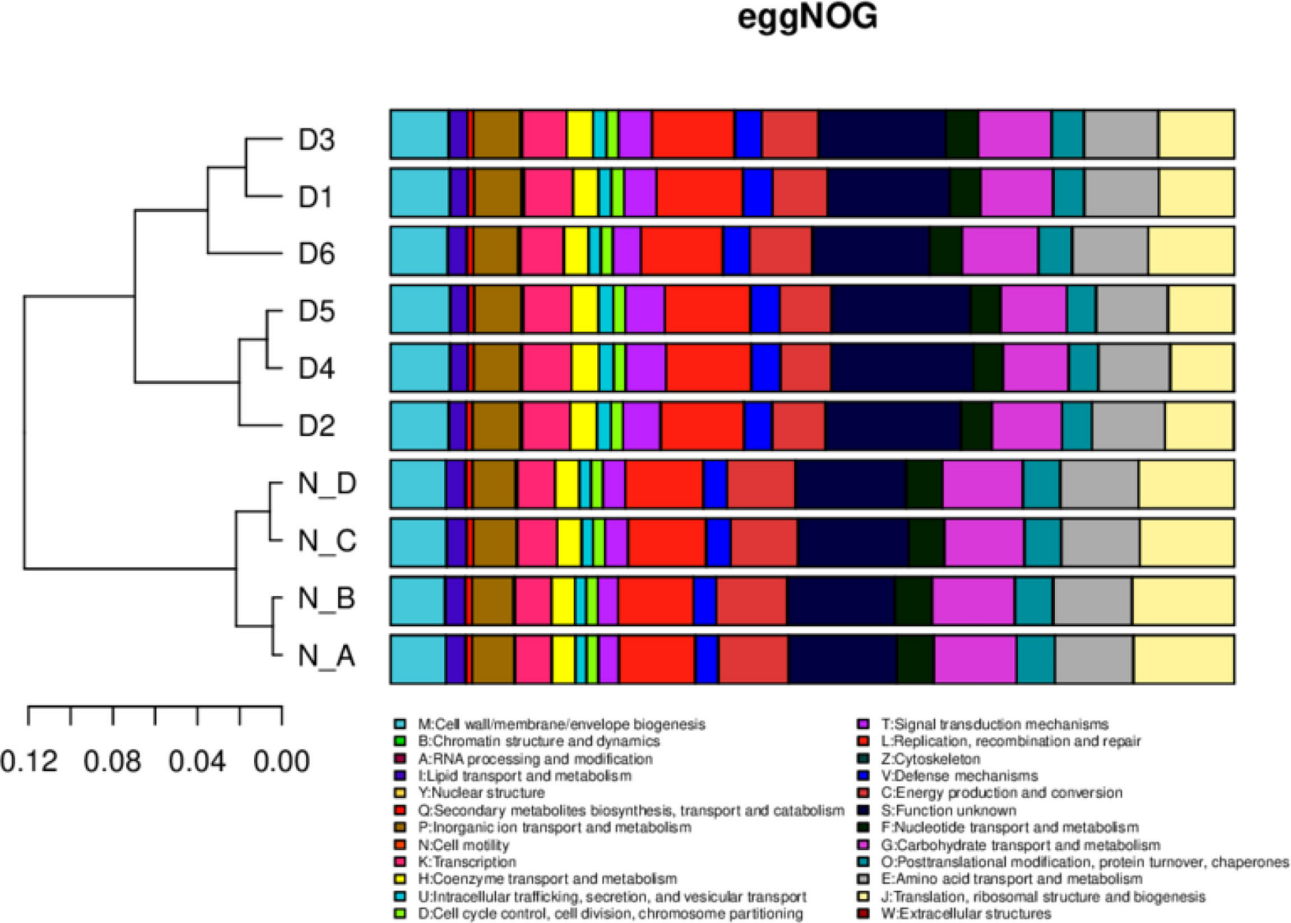
The functional relative abundance analysis. eggNOG. D1, D2, D3, D4, D5, and D6 represent the diarrheal samples; N_A, N_B, N_C, and N_D represent the normal samples.

**Figure 8:**
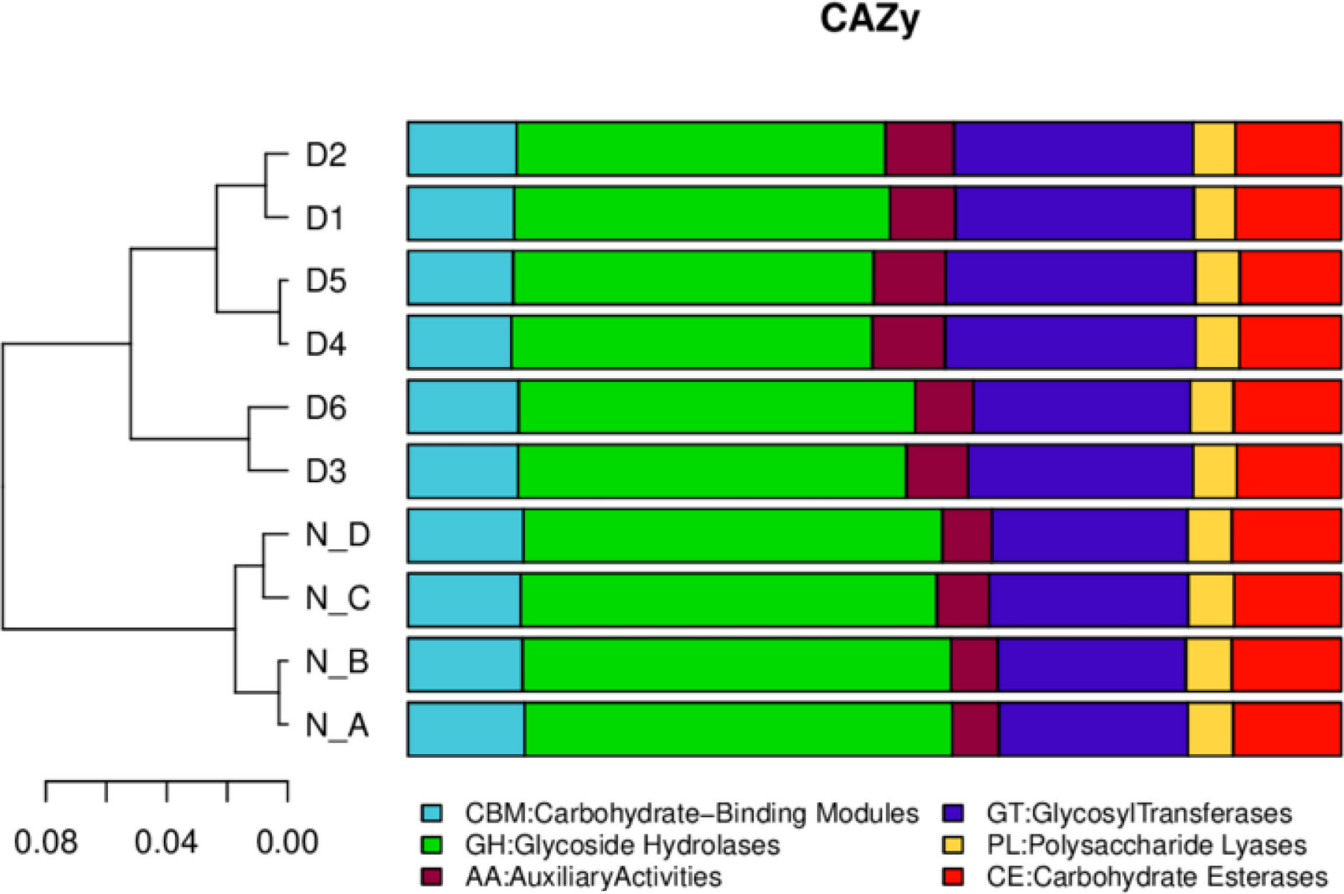
The functional relative abundance analysis. CAZy. D1, D2, D3, D4, D5, and D6 represent the diarrheal samples; N_A, N_B, N_C, and N_D represent the normal samples.

In eggNOG, about 24 cell metabolic pathways, i.e. wall biogenesis, chromatin structure and dynamics, were reported in all animals. In secondary metabolites biosynthesis, signal transduction mechanisms were non significantly higher in diarrheal animals, while translation, ribosomal structure and biogenesis were higher in healthy yaks (**Figure 7**). In CAZy, carbohydrate-binding modules, glycosyltransferases, glycoside hydrolases, polysaccharide lyases, auxiliary activities and carbohydrate esterase were found in both groups. Moreover, glycoside hydrolases were found higher in normal yaks, while glycosyltransferases were higher in diarrhea yaks non significantly (**Figure 8**).

**Figure 9:**
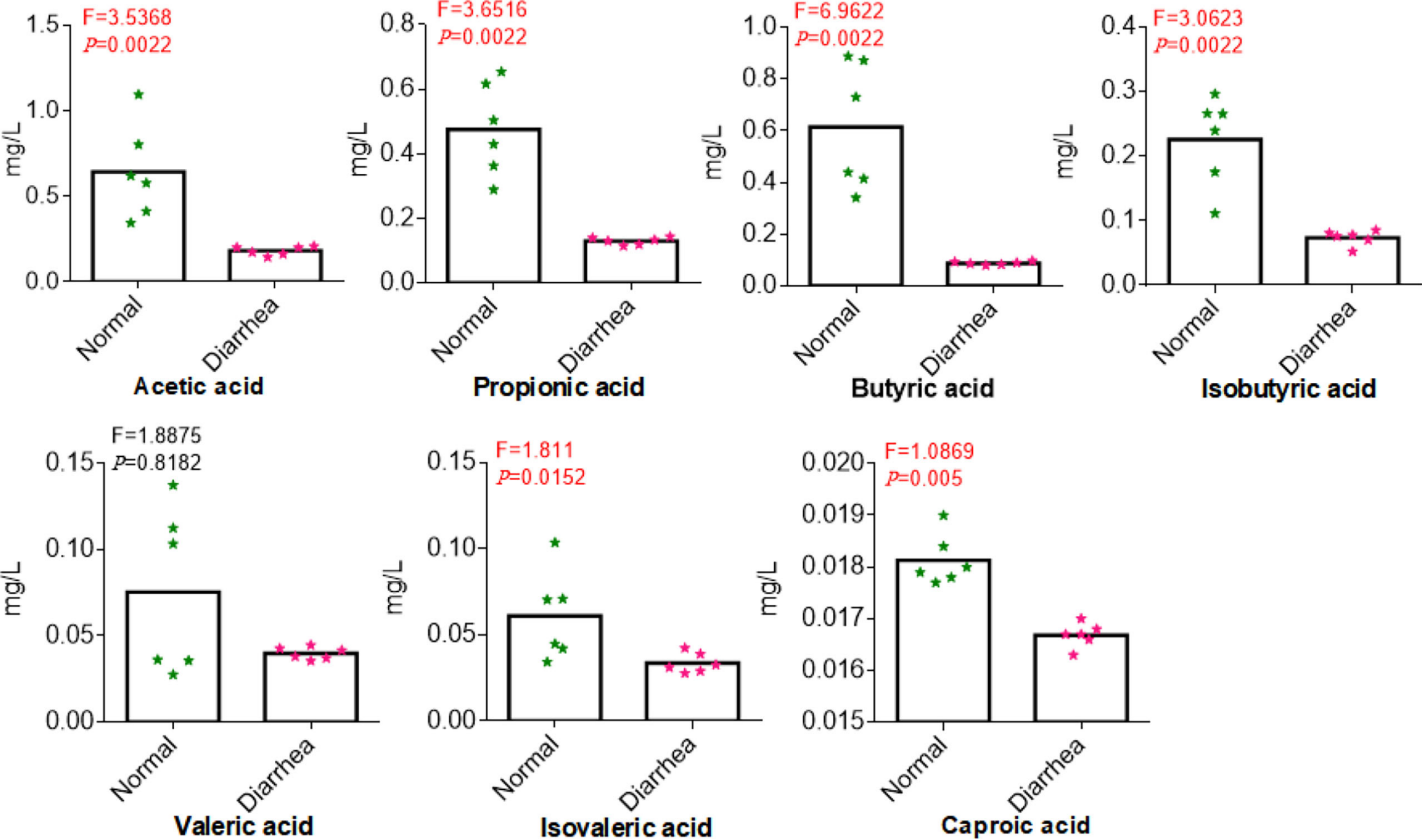
Comparing the concentrations of SCFA in normal and diarrhea groups. The *x*-axis corresponds to SCFA, and the *y*-axis corresponds to the concentration of SCFA.

**Figure 10:**
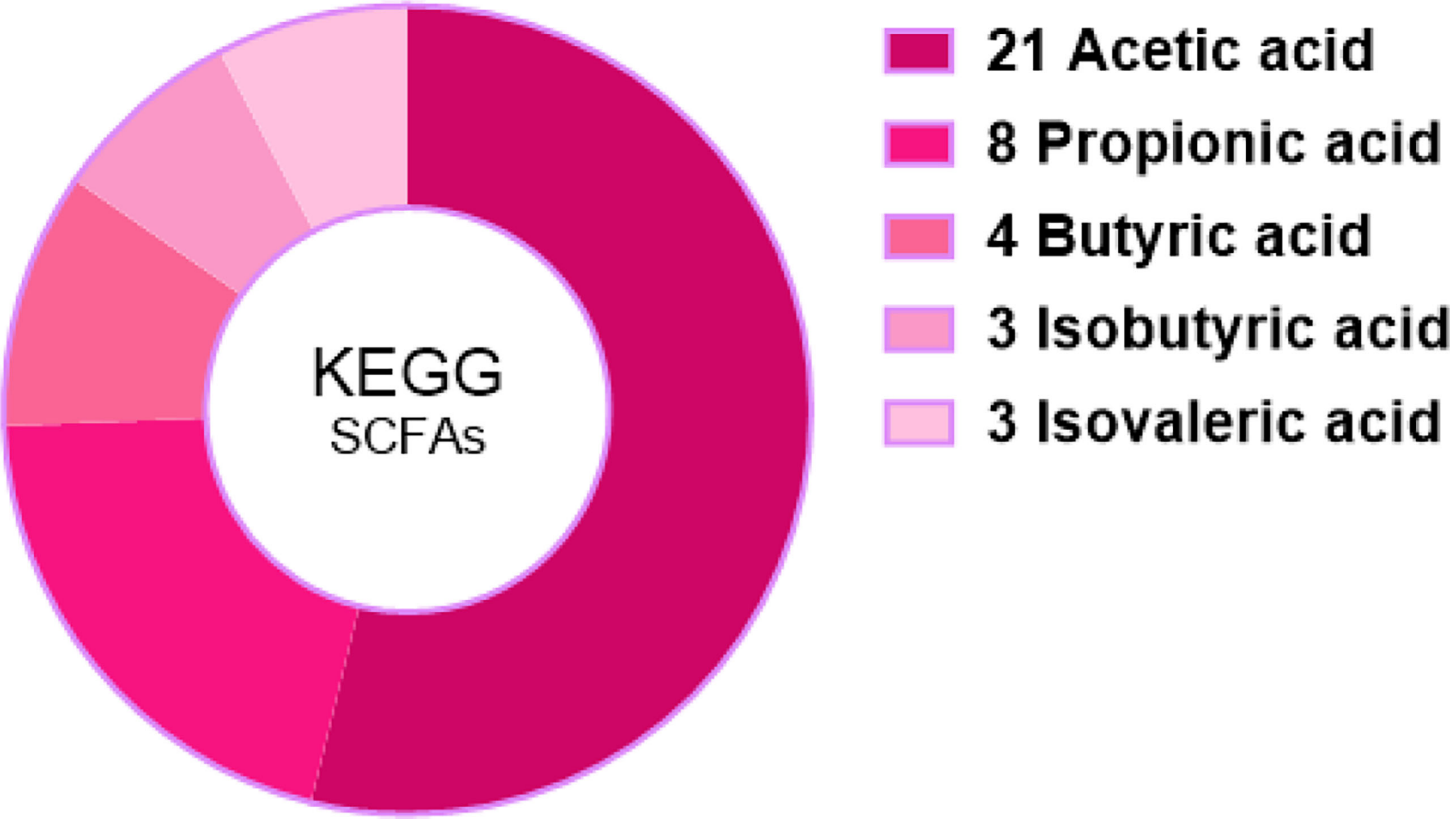
Statistic analysis of SCFA-relevant KEGG signal pathways in the intestinal metabolism through kanehisa laboratories.

Among 366,163 significant differential abundance genes,141,305 were upregulated and 224,858 were downregulated in diarrheal yaks compared with normal yaks *via* DESeq analysis (**Supplementary Figure S16A**). Metagenomics binning analysis with bin 33 (Bacteroidales) (*p* < 0.05) was significantly higher in diarrheal animals, while bin 10 (*p* < 0.0001), bin 30 (Clostridiales) (*p* < 0.05), bin 51 (Lactobacillales) (*p* < 0.05), bin 8 (Lachnospiraceae) (*p* < 0.05), and bin 47 (Bacteria) (*p* < 0.05) was significantly higher in normal animals (**Supplementary Figures S17, S18**). In the current study, 6 out of 7 SCFA were uncovered significantly lower in diarrheal yaks from 100 mixed fecal samples by employing GC-MS/MS (*p* < 0.05) (**Figure 9**). Statistical analysis showed that SCFA acetic acid (53.8%) accounted for most of the acetate (50%–70%) in the intestine, which represents the major KEGG signal pathway (**Figure 10**).

DESeq analysis was employed to uncover significant differential abundance genes between two yak groups at fold change ≥2 and *p*-value <0.01 (Anders and Huber, 2010). There were 366,163 significantly essential differential abundance genes in diarrheal yaks compared with normal yaks, with 141,305 upregulated and 224,858 downregulated (**Supplementary Figure S16A**). Differential abundant genes were compared against the cluster of orthologous protein database. Most of the genes were related to amino acid metabolism, replication, recombination, cell wall biogenesis, carbohydrate transportation and metabolism, translation, ribosomal structure, and biogenesis (**Supplementary Figure S16B**). Annotation of abundant differential genes in the KEGG path showed the relationship of the gene with metabolism (**Supplementary Figure S19A**). Enrichment analysis of abundant differential genes in the KEGG pathway revealed that 24 genes showed a significant performance, such as carbohydrate metabolism gene, the global and overview map gene, the amino acid metabolism gene, etc. The enrichment factor in the *x*-axis represented the significant enrichment level of differentially expressed abundant genes. Lipopolysaccharide biosynthesis was at the highest level of differentially expressed abundant genes (**Supplementary Figure S19B**). Most significantly, different genes were related to the ribosomes (*p* < 0.001), peptidoglycan biosynthesis (*p* < 0.001), and homologous recombination (*p* < 0.001).

### Binning Analysis of the Metagenome of Intestinal Microbiota in Yaks

Metagenomics binning analysis revealed 9 bins with genome completeness >50% and contamination rate <10% in yaks (**Table 3**). The genome completeness was 93.10% to 99.31% containing Bacteria, Selenomonadales, Clostridiales, Firmicutes, Lactobacillus, and Bacteroidetes. Through heat map of 76 bins, bin 33 (*p* < 0.05) was significantly higher in the diarrheal group, while bin 10 (*p* < 0.0001), bin 30 (*p* < 0.05), bin 51 (*p* < 0.05), bin 8 (*p* < 0.05), and bin 47 (*p* < 0.05) was significantly higher in the normal group (**Figure 5**; **Supplementary Figure S20**). The genomics of those bins were Bacteroidales (bin 33), Clostridiales (bin 30), Lactobacillales (bin 51), Lachnospiraceae (bin 8), and Bacteria (bin 47).

**Table 3:** Metagenomics binning analysis of yak intestine microbiota *via* Maxbin2 and Maxbat2.

Binner	Bin	Completeness (%)	Contamination (%)	GC	Lineage	N50	Size (bp)
Metabat2	bin.17	99.31	1.284	0.348	Bacteria	54,875	2,180,769
	bin.34	95.48	3.347	0.451	Selenomonadales	8,117	1,893,381
	bin.64	93.03	2.369	0.419	Clostridiales	11,985	1,727,329
Maxbin2	bin.44	97.13	0.692	0.429	Clostridiales	18,616	1,834,347
	bin.38	96.84	0.693	0.394	Selenomonadales	37,174	1,818,504
	bin.21	96.81	2.259	0.318	Firmicutes	11,181	2,115,861
	bin.2	95.04	1.436	0.384	Lactobacillus	18,617	1,934,486
	bin.41	93.85	6.704	0.489	Bacteroidetes	11,868	1,965,257
	bin.58	93.10	1.006	0.450	Clostridiales	21,746	1,436,082

### Quantitative Analysis of SCFA in Yaks

Sample quality control analysis showed that the TIC from different samples nearly overlapped completely, which indicated that the current data were repeatable and reliable (**Supplementary Figure S20A**). The TIC from yak mixture samples showed several single waves without overlapping, which revealed valid results of SCFA (**Supplementary Figure S20B**). In the present study, all the correlation coefficients (*R* ^2^) of each equation of linear regressions were over 0.994≈1.000, which ensured the accuracy of SCFA values (**Table 4**).

**Table 4:** Equation of linear regressions detected by standard samples *via* GC-MS.

SCFA	Equation	*R* ^2^	Linearity range (mg/L)
Acetic acid	*y* = 10.001982 * *x* − 3.631493	0.998242	0.1–20
Propionic acid	*y* = 4.991172 * *x* − 1.707163	0.995486	0.1–9.5
Isobutyric acid	*y* = 9.838497 * *x* − 1.317041	0.998669	0.1–8
Butyric acid	*y* = 69.122314 * x-21.545157	0.997229	0.1–20
Isovaleric acid	*y* = 72.284002 * *x* − 14.156633	0.998779	0.1–20
Valeric acid	*y* = 43.669973 * *x* − 11.692827	0.998961	0.1–20
Caproic acid	*y* = 34.683801 * *x* − 7.396451	0.994521	0.1–8

A significant difference of the seven SCFA except valeric acid was found between normal and diarrheal yaks. Acetic acid, propionic acid, butyric acid, isobutyric acid, and caproic acid were found in normal yaks, significantly higher than diarrheal yaks (p-value <0.01). Isovaleric acid in normal yaks was also significantly higher than diarrhea yaks (p-value <0.05) (**Figure 9**).

## Discussion

In Norway, about US$10 million loss was noted due to calves death affected by diarrhea in 2006 (Østerås et al., 2007). As an agricultural country, the development of animal husbandry is important, especially in Hongyuan (China), like plateau areas. In our study, the prevalence of diarrhea in yaks was estimated at about 15%–25% and 5%–10% in calves and adults, respectively (**Table 1**). Diarrhea in yaks was significantly higher in yak calves (**Supplementary Figure S2**), which was in line with the widely accepted knowledge that morbidity of diarrhea in calves is more serious (Dore et al., 2019). Therefore, discovering the potential causes of this emerging diarrhea is urgent and meaningful, especially on the remote plateau.

The intestinal microbiota is also considered an additional organ, which comprises billions of microorganisms. The intestinal microbiota is important in the synthesis and metabolism of nutrients, hormones, and vitamins, playing a role in drug utilization, pathogen fortification, and immune system maturation (Blaut, 2015; Mangiola et al., 2016; Rinninella et al., 2019). Therefore, the imbalance of intestinal microbiota may lead to serious diseases. Previously, we performed high-throughput sequencing of intestinal microflora from diarrheal yaks. Our study found 41 genera of bacteria in perinatal healthy yaks, while 145 genera of bacteria were only tested in healthy perinatal yaks (Han et al., 2017). Moreover, 212 genera of fungus were found in healthy yaks, and 373 and 208 genera of fungus were found in calves and diarrheal adult yaks, respectively (Li et al., 2018). However, 16s RNA sequencing was limited to the genus level. In the current study, metagenomics sequencing was employed to explore the potential pathogens of diarrhea in yaks. Violin box plot also showed the higher gene abundance in diarrhea yaks in concern with the degree of dispersion than normal yaks (**Supplementary Figure S4**). Such results may predict the different microbiota compositions in diarrheal and normal animals. We found more significantly lower species composition in diarrheal yaks (**Supplementary Figure S5**).

*Staphylococcus aureus* is a commonly known bacterium related to human and animal foodborne diseases (Kroning et al., 2020). This pathogen also causes orthopedic implant-associated infection, especially methicillin-resistant bacteria (Czuban et al., 2020). As infected animals are commonly treated with antimicrobial agents, thus serious antimicrobial resistance is becoming a public health concern worldwide (Kroning et al., 2020). Diseases such as gastroenteritis, nausea, vomiting, abdominal cramps, etc., are usually seen in infected individuals (Wang et al., 2020). The increase of *Staphylococcus aureus* in diarrheal yaks may indicate a potential threat for local herdsmen. *Bacteroides coprophilus* was previously reported as proinflammatory in ankylosing spondylitis (Zhou et al., 2020), which may infer with an inflammatory status of diarrhea yaks. *Bacteroides plebeius* was previously found significantly higher in type 2 diabetes mellitus patients (Wang et al., 2020), also regarded as a biomarker of this disease. Thus, the increase of *Bacteroides plebeius* in diarrhea animals means the abnormal glucose metabolism in yaks.

The butyrate-producing bacteria *Butyricicoccus pullicaecorum* is commonly linked with inflammatory conditions of the intestinal ecosystem (Andrade et al., 2020), which may cause inferred inflammatory response during diarrhea in yaks. Though *Babesia ovata* is a low pathogenic species, its infection may lead to severe damage in cattle when coinfected with *Theileria orientalis* (Nguyen et al., 2019). A previous study reported that the prevalence of *T. orientalis* in yaks was 9.7% on the plateau (Li et al., 2017). The infection of *T. orientalis* may be the main reason for bloody diarrhea in yaks (**Supplementary Figure S1**). *Fusobacterium mortiferum* was usually isolated from Crohn’s and Behcet’s patients (Garcia-Saura et al., 2019). Also, *Ruminococcus gnavus* is a Crohn’s disease-associated pathobiont (Yu et al., 2020), which was in line with the diarrhea symptoms in yaks. *Anaplasma phagocytophilum* is a commonly reported emerging tick-borne zoonotic pathogen causing anaplasmosis (Adamska, 2020). This bacterium primarily infects host neutrophils, which break the first-line immune defensive barrier in mammalians (Tang et al., 2020). The infected animals show typically anemia (Langenwalder et al., 2020), which reveal that *A. phagocytophilum* may contribute to diarrhea in yaks. *Bacteroides fluxus* is a pathogenic species of *Bacteroides* that displays numerous and high rates of antibiotic resistance. Higher abundance of *Bacteroides fluxus* means this bacterium plays a potential role in diarrhea. *Firmicutes bacterium* was associated with lipogenesis metabolism in animals with nonalcoholic fatty liver disease (Paul et al., 2019). The increased *Firmicutes bacterium* (CAG:424) in diarrheal yaks may cause dyslipidemia. Bovine viral diarrhea and Rotavirus were also reported in yaks (Li et al., 2018; Yan et al., 2020), which could infer that the increased abundance of these viruses may cause diarrhea in yaks.

*Fournierella massiliensis* is a new human-associated member of the family Ruminococcaceae (Togo et al., 2017), which may have little relationship with diarrhea. *Bacteroides vulgatus* was the main cause of polycystic ovary syndrome through disrupted ovarian functions and aggravated insulin resistance (Qi et al., 2019). This means increment of *Bacteroides vulgatus* in yaks may cause diarrhea *via* affecting glycol metabolism. *Klebsiella pneumonia* causes many infections, i.e., pneumonia, urinary tract infection, meningitis, and bacteremia (Furlan et al., 2020), which indicates the infection status of diarrheal yaks. While *Firmicutes bacterium* CAG:110 (*p* < 0.01), *Clostridiales bacterium* (*p* < 0.001), *Ruminococcaceae bacterium* (*p* < 0.001), *Clostridium* sp. CAG:413 (*p* < 0.001), *Clostridia bacterium* (*p* < 0.001), *Firmicutes bacterium* CAG:137 (*p* < 0.001), *Methanobrevibacter olleyae* (*p* < 0.001), *Bacteroidales bacterium* WCE2008 (*p* < 0.001), *Ruminococcus flavefaciens* (*p* < 0.001), *Methanobrevibacter ruminantium* (*p* < 0.001), *Bacteroidete bacterium* (*p* < 0.001), *Anaerotruncus* sp. Cag:390 (*p* < 0.001), *Clostridium* sp. CAG:448 (*p* < 0.001), *Firmicutes bacterium* (*p* < 0.01), *Firmicutes bacterium* CAG:124 (*p* < 0.001), and *Firmicutes bacterium* CAG:170 (*p* < 0.001) were significantly lower (**Figure 8** **C**). *Firmicutes bacterium* CAG:110 was found to be potentially associated with swine feed efficiency variation in cecum microbiota *via* the utilization of dietary polysaccharides and dietary protein (Quan et al., 2020). It means the dropped abundance of *Firmicutes bacterium* CAG:110 decreases feed efficiency. *Firmicutes bacterium* CAG:137, *Firmicutes bacterium* (*p* < 0.01), *Firmicutes bacterium* CAG:124 (*p* < 0.001), and *Firmicutes bacterium* CAG:170 (*p* < 0.001) belong to host energy uptake or storage-limiting related *Firmicutes*, which are one of the most abundant bacteria in animals and human beings (Ley et al., 2005). The lower of *Firmicutes bacteria* may linked to glycol metabolism, which may further cause diarrhea.

Butyrate-producing *Clostridiales bacterium* is associated with protecting the host from colorectal cancer, immune, and metabolic disorders (Pichler et al., 2020). It means dropped *Clostridiales bacterium* in yaks may contribute to diarrhea. *Ruminococcus flavefaciens* works with noncellulolytic *Treponema* or *Butyrivibrio* species that can accelerate cellulose digestion (Cheng et al., 1991). The lower *Ruminococcus flavefaciens* in diarrheal yaks may decrease the cellulose efficiency. Previously, more deficient *Ruminococcaceae bacterium* was found in hospitalized patients, cirrhosis (Paul et al., 2019), and diarrhea foals (Schoster et al., 2017). The deceased of this bacterium may provide an insight that *Ruminococcaceae bacterium* has a relationship with diarrhea in yaks. CAG:413, CAG:448 and *Clostridia bacterium* belonged to the *Clostridium* genus, recognized as a beneficial bacteria to the host (Guo et al., 2020). The lower of this three *Clostridium* spp. may promote diarrhea in yaks. *Methanobrevibacter olleyae* and *Methanobrevibacter ruminantium* composed the *M. ruminantium* clade, which belongs to the ruminant *Methanobrevibacter* genus (Kelly et al., 2016). These two bacteria with other *Methanobrevibacter* spp. compose the rumen methanogenic community (Danielsson et al., 2012), which indicates that diarrheal yaks also have decreased methane production. *Bacteroidales bacterium* WCE2008 is a *Bacteroidales* species accepted as “beneficial” microbes (Anderson, 1996). A previously dropped abundance of *Bacteroidales* was found in pediatric patients with CD (Anderson, 1996), which may infer that the imbalance of this bacterium is related to diarrhea in yaks. Higher abundance of *Bacteroidete bacterium* is related to healthy lean of host, as it can generate three main SCFA, butyrate, acetate, and propionate (Davis, 2018). The decreased *Bacteroidete bacterium* in animals contribute to diarrhea. *Anaerotruncus* can utilize cheese whey to produce acetic and butyric acids (Gao et al., 2016). The decreased *Anaerotruncus* sp. CAG:390 in diarrhea may affect fatty acid metabolism in ruminants.

Microbiota is a key regulator of digestion, extraction, synthesis, and absorption of many nutrients and metabolites, i.e., bile acids, lipids, amino acids, vitamins, and SCFA (Rinninella et al., 2019). The SCFA are not only principal nutrient substrates of intestinal epithelial cells but also can regulate the epithelial barrier (Mazzawi et al., 2019). Previously, concentrations of SCFA was related with diarrhea-predominant irritable bowel syndrome patients (Mazzawi et al., 2019). SCFA could mitigate adenine-induced chronic kidney disease (Mikami et al., 2020); preoperative fecal levels of SCFA had an important impact on the occurrence of postoperative infectious complications in patients with esophageal cancer (Motoori et al., 2020). SCFA in fecal samples was commonly used to approximate gut levels, which can infer the relationship between intestinal SCFA production and fecal levels (Haenen et al., 2013). It reveals that the imbalance of gut microbiota dropped the levels of SCFA in diarrhea, so it can be supposed that SCFA has immunological and regulatory functions (Farup et al., 2016), activating anti-inflammatory signaling *via* acting as ligands of G-protein-coupled receptors, e.g., GPR109A, GPR41, and GPR43 (Parada Venegas et al., 2019). The current results are in line with diarrhea-dominant IBS with lower levels of SCFA (Huda-Faujan et al., 2010). Among the common SCFA, acetate (C2), propionate (C3), and butyrate (C4) are the most in number, produced by anaerobic fermentation of dietary fibers in the intestine (Parada Venegas et al., 2019). Those three SCFA accounted for 90% of SCFA produced by gut microbiota, which depict the beneficial effects on intestinal epithelial cells and immune cells in the intestinal mucosa (Rechkemmer and Vonengelhardt, 1988; Chen et al., 2020).

Acetate was reported to mediate joint inflammation in a murine gout model *via* inflammasome assembly and IL-1β (Vieira et al., 2015). Propionic acid was found to be increased in gut-associated Treg cells (relates to systemic immune reaction and disease amelioration) (Duscha et al., 2020). Butyrate is a primary energy source for colonocytes and can maintain intestinal homeostasis through anti-inflammatory actions *via* inhibiting nuclear factor-kappa β and histone deacetylation by promoting epithelial barrier function (Morrison and Preston, 2016; Parada Venegas et al., 2019). Previously, a lower abundance of butyrate-producing bacteria and fecal butyrate were found in stroke patients as higher risk factors (Zeng et al., 2019). *Bacteroidetes* from *Firmicutes* mainly produce acetate and propionate, while butyrate is mainly produced by phylum *Firmicutes*, i.e., *Faecalibacterium prausnitzii*, *Clostridium leptum*, *Eubacterium rectale*, and *Roseburia* spp (Louis et al., 2010).. In a previous study, *Firmicutes* phylum was found clearly lower in diarrheal yaks (*p* < 0.05) (Han et al., 2017). Genus of *Clostridium*_IV (*p* < 0.01) and *Clostridium*_XI (*p* < 0.05) were found significantly lower in diarrheal yaks except *Clostridium* XVIII (*p* < 0.01). The genera of *Bacteroides* (*p* < 0.05) and *Faecalibacterium* (*p* < 0.05) were found significantly higher in diarrhea yaks, while no significant difference was found in the genera of *Eubacterium*, *Eubacterium*, and *Roseburia* (Han et al., 2017). However, among all those genera, *Clostridium*_IV and *Clostridium*_XI were the dominant (Han et al., 2017), which may uncover that the decreasing of clostridium may cause the drop of SCFA (C2-C4). In the current study, isobutyric acid, isovaleric acid, and caproic acid were found significantly lower in diarrheal animals (*p* < 0.05), which was in line with patients suffering from cirrhosis and neuromyelitis optica spectrum disorders (Gong et al., 2019; Jin et al., 2019). As SCFA plays a critical role in mucosal integrity and immune response (Wang et al., 2019). So, the dropping of SCFA (C4-C6) may damage mucosal and inflammation response. SCFA generation bacteria of *Anaerotruncus* sp. CAG:390, *Clostridiales bacterium* and *Butyricicoccus pullicaecorum* are lower in number in diarrheal yaks. Although *Fusobacterium mortiferum* producin*g* butyric and acetic acids increase significantly (Thompson et al., 1992). However, it could not affect the dropping trend of SCFA in diarrheal animals. Moreover, the abundance of SCFA acetic acid (53.85%) in the current study was the primary level of acetate (50%–70%) in the intestine (Lavelle and Sokol, 2020).

## Conclusion

In conclusion, we estimated the prevalence of emerging diarrhea in yak calves was 15%–25% and 5%–10% in adult yaks. Besides the high prevalence of *Staphylococcus aureus*, *Babesia ovata*, *Anaplasma phagocytophilum*, *Bacteroides fluxus*, viruses, *Klebsiella pneumonia*, and inflammation-related bacteria, the decrease of SCFA was observed in diarrheal yaks. Our results will give insights into the prevention and treatment of emerging diarrhea issue in yaks on the plateau.

## Data Availability Statement

The datasets presented in this study can be found in online repositories. The names of the repository/repositories and accession number(s) can be found in the article/**Supplementary Material**.

## Ethics Statement

Ethical review and approval were not required for the animal study because only fresh faecal samples were collected from diarrheal animals so no ethical permission was required. Written informed consent was obtained from the owners for the participation of their animals in this study.

## Author Contributions

KL, JiL, and DQ designed the research. KL, ZZ, JuL, LP, YW, and AL conducted the experiments. KL analyzed the data. KL and MS prepared the manuscript. MK revised and finalized the manuscript. All authors listed have made a substantial, direct, and intellectual contribution to the work and approved it for publication.

## Funding

The current research was supported by the China Postdoctoral Science Foundation (2020M672378), China Agriculture Research System (CARS-37), and Tibet Autonomous Region Science Fund (ZDZX2018000043).

## Conflict of Interest

The authors declare that the research was conducted in the absence of any commercial or financial relationships that could be construed as a potential conflict of interest.

## Publisher’s Note

All claims expressed in this article are solely those of the authors and do not necessarily represent those of their affiliated organizations, or those of the publisher, the editors and the reviewers. Any product that may be evaluated in this article, or claim that may be made by its manufacturer, is not guaranteed or endorsed by the publisher.
