# Supplementary material for: Effects of Short-Chain Fatty Acid Modulation on Potentially Diarrhea-Causing Pathogens in Yaks Through Metagenomic Sequencing
Source: Front Cell Infect Microbiol. 2022 Mar 23;12:805481. doi: 10.3389/fcimb.2022.805481 (PMC8983862; doi:10.3389/fcimb.2022.805481)
Supplement: Supplementary file 1 [file DataSheet_1.docx]

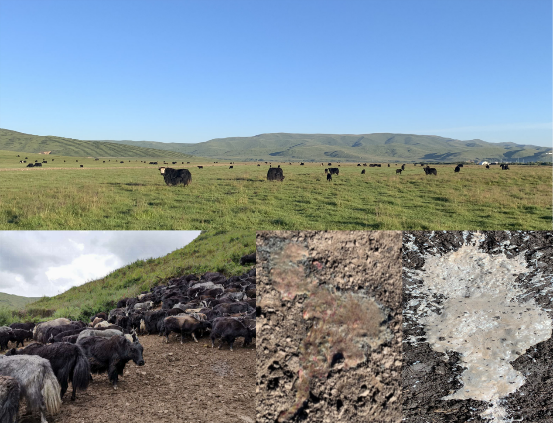


**Figure S1.** Diarrhea in yaks on the high plateau.


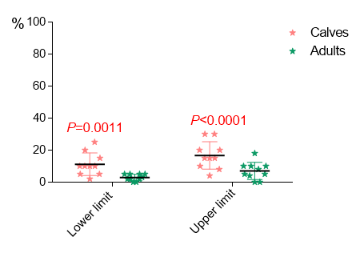


**Figure S2.** Comparing the prevalence of diarrhea in yaks in different farms (As all the prevalence data were estimated, we used upper limit prevalence and lower limit prevalence for comparing, respectively.


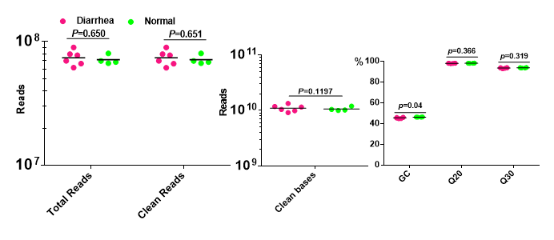


**Figure S3.** Statistics of sequencing data of yak microbiota samples.


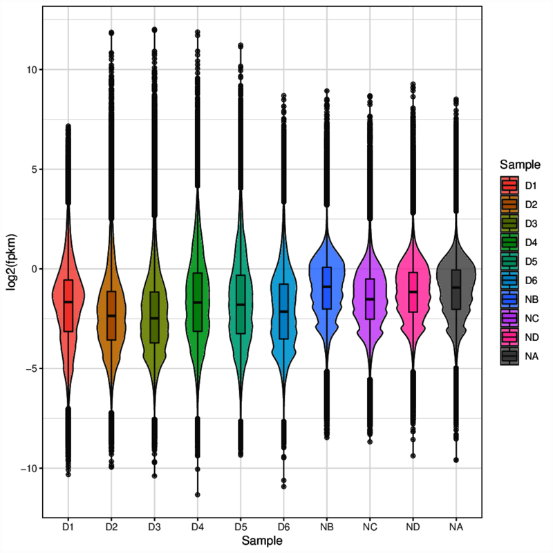


**Figure S4.** Gene abundance distribution of each yak samples.


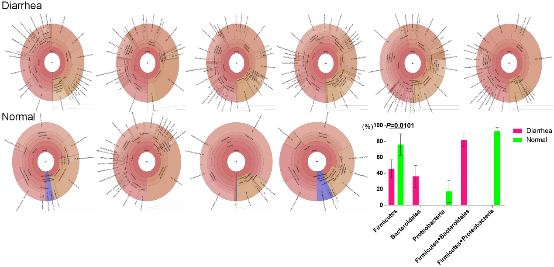


**Figure S5.** Annotated analysis of different yak microbiota species abundance via Krona.


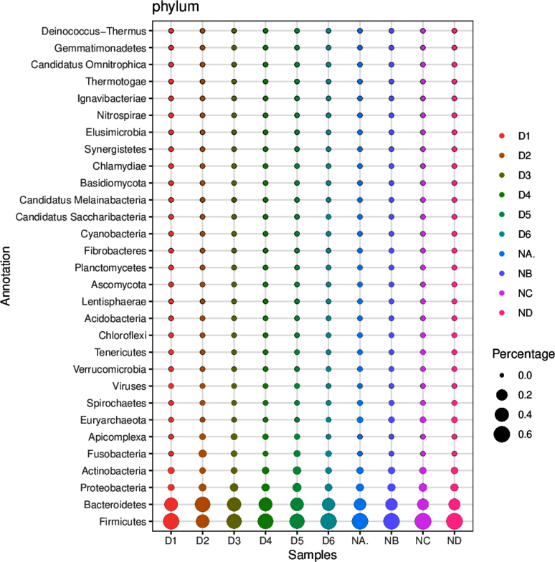


**Figure S6.** Intestinal microbiota structure of yaks in Phylum level.


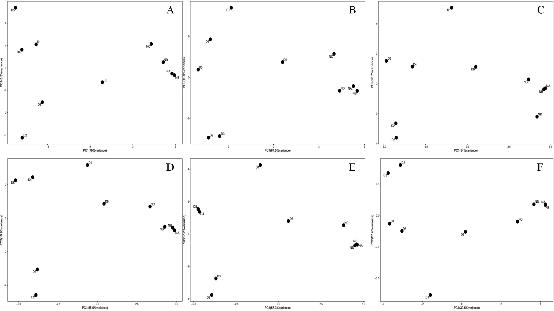


**Figure S7. A:** Principal component analysis of Intestinal microbiota of yaks in Phylum level. **B**: Principal component analysis of Intestinal microbiota of yaks in Class level. **C**: Principal component analysis of Intestinal microbiota of yaks in Order level. **D**: Principal component analysis of Intestinal microbiota of yaks in Family level. **E**: Principal component analysis of Intestinal microbiota of yaks in Genus level. **F**: Principal component analysis of Intestinal microbiota of yaks in Species level.


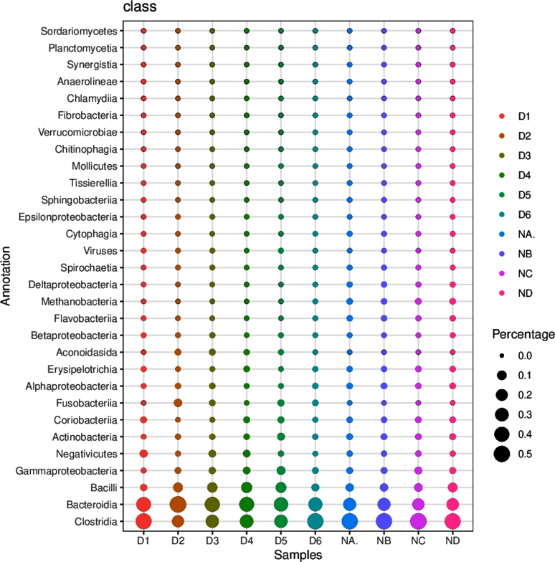


**Figure S8.** Intestinal microbiota structure of yaks in Class level.


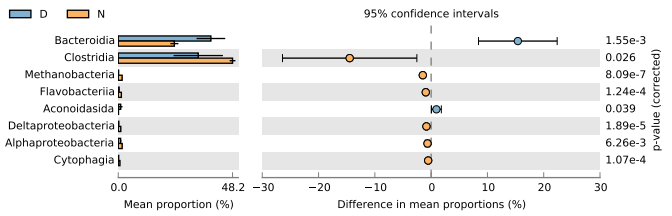


**Figure S9** Comparing Intestinal microbiota difference between different yaks in Class level. D, diarrheal group samples; N, normal group samples.


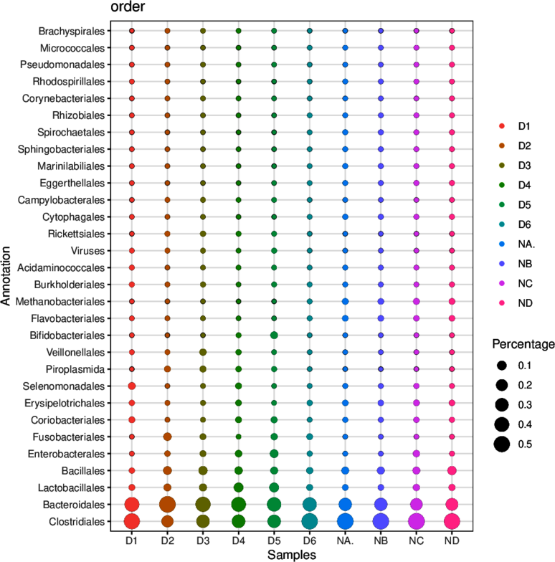


**Figure S10** Intestinal microbiota structure of yaks in Order level.


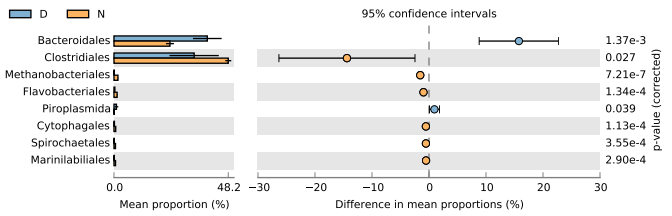


**Figure S11** Comparing Intestinal microbiota difference between different yaks in Order level. D, diarrheal group samples; N, normal group samples.


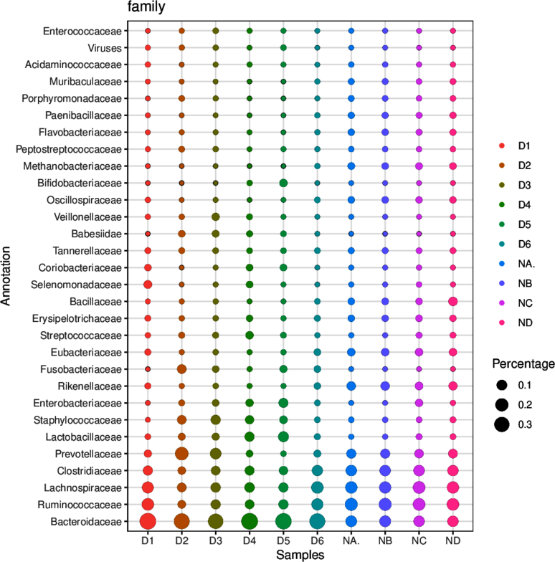


**Figure S12** Intestinal microbiota structure of yaks in Family level.


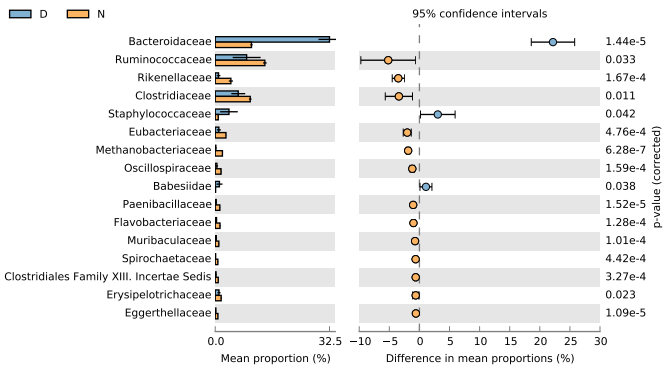


**Figure S13** Comparing Intestinal microbiota difference between different yaks in Family level. D, diarrheal group samples; N, normal group samples.


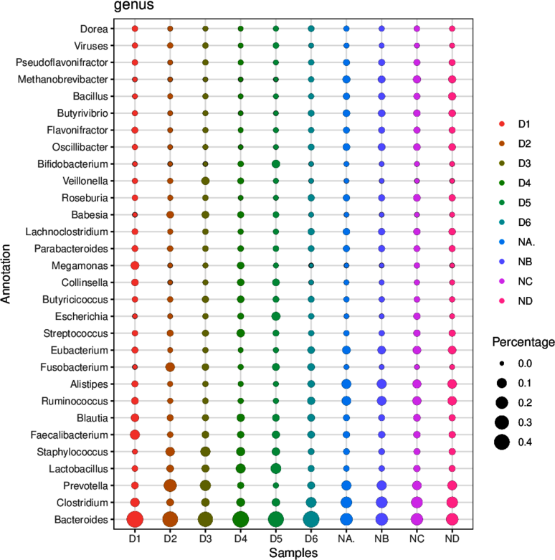


**Figure S14** Intestinal microbiota structure of yaks in Genus level.


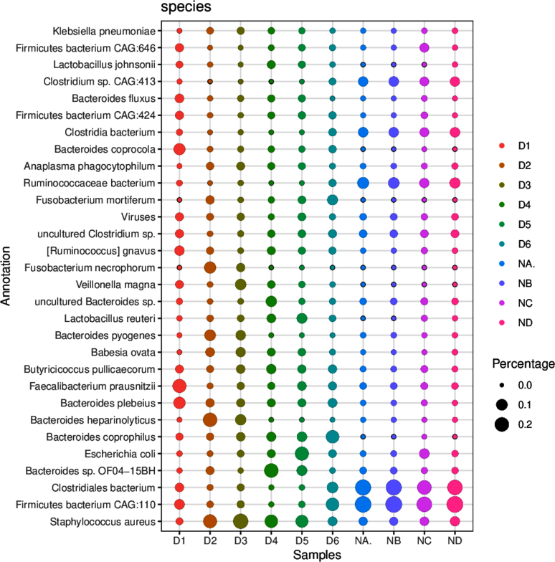


**Figure S15** Intestinal microbiota structure of yaks in Species level.


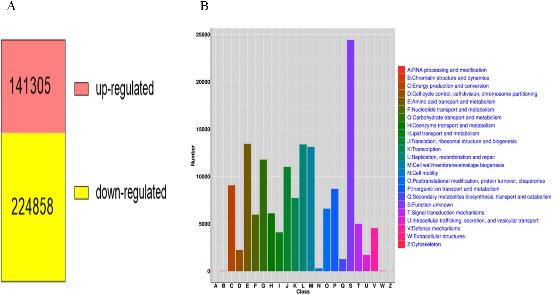


**Figure S16 A:** Comparing the differential abundance genes in the two yak groups **B:** Classification and statistics of differential abundance genes in COG.


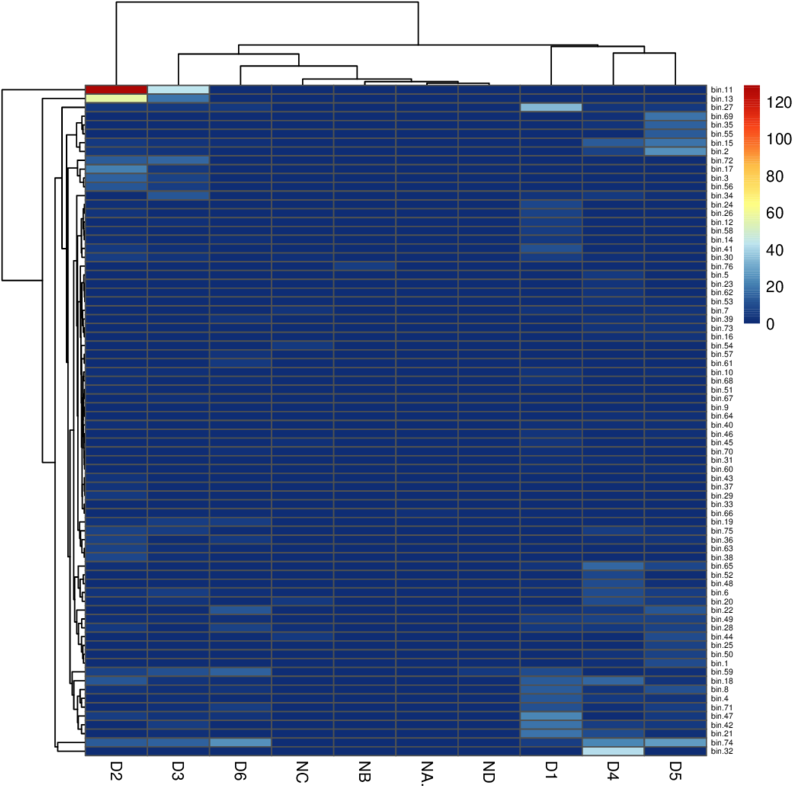


**Figure S17** Heat map analysis of the top 76 bins in different yaks. D1, D2, D3, D4, D5, D6 represented diarrheal samples; NA, NB, NC, ND represented normal samples.


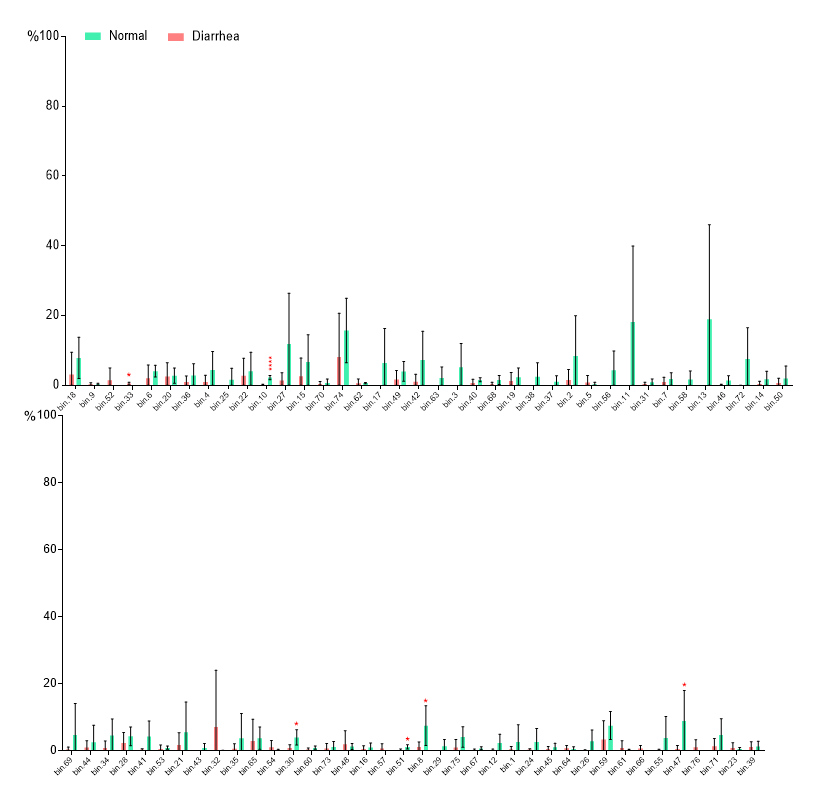


**Figure S18** Comparing of different bin related species abundance in the two yak groups.


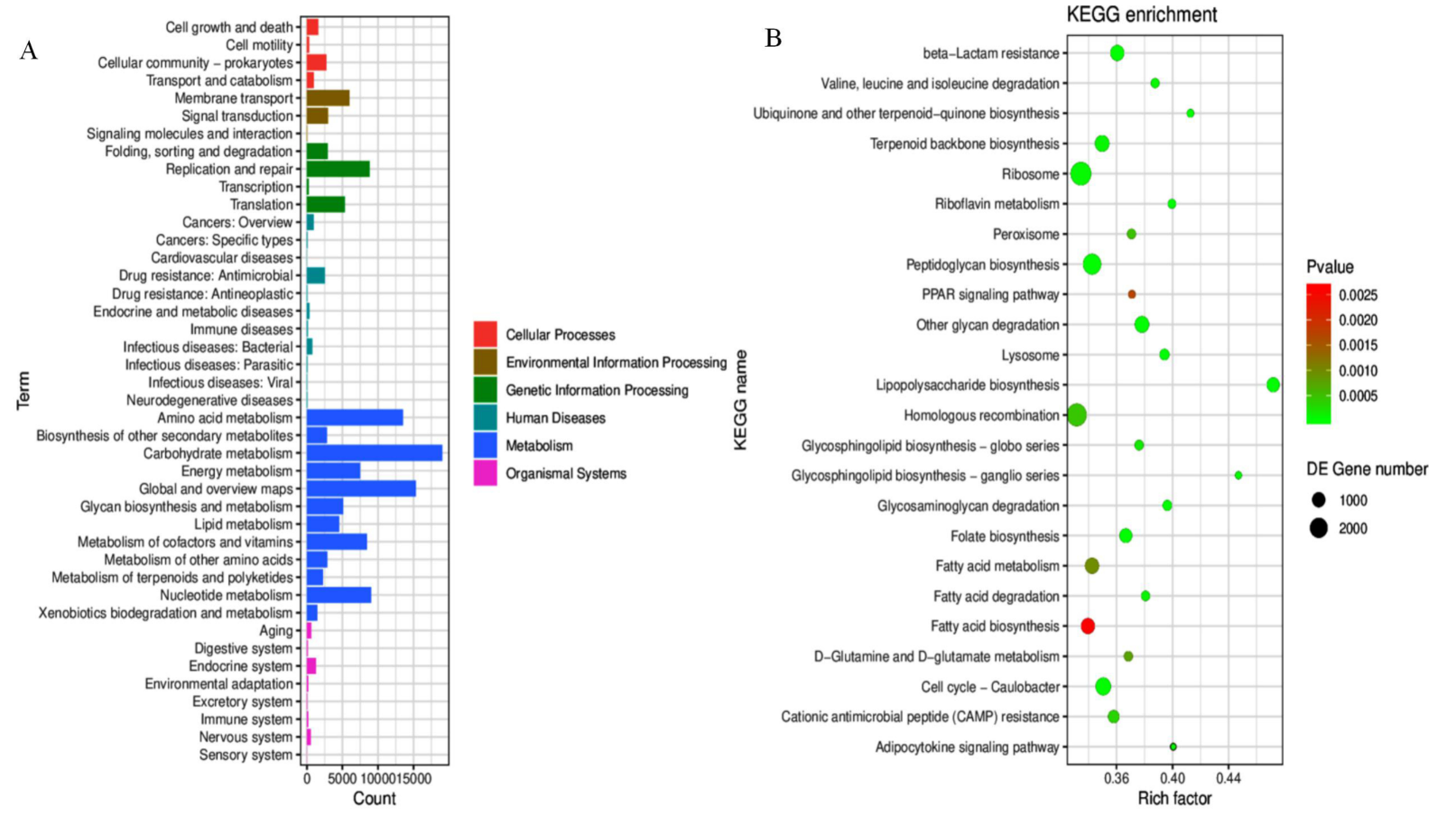


**Figure S19 A:** Annotation of differential abundance genes in KEGG path. **B:** Enrichment analysis of differential abundance genes in KEGG pathway.


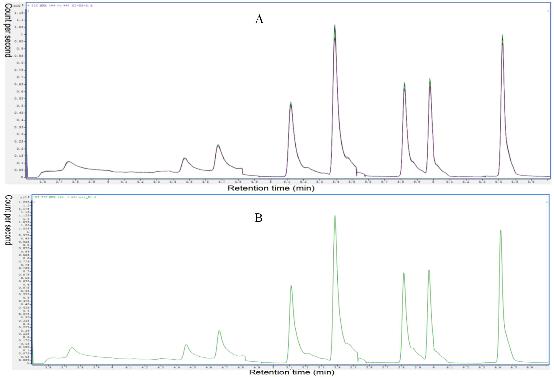


**Figure S20 A:** Overlap figure of total ions current of quality control samples analyzed by Agilent MassHunter. The X-axis corresponds to the retention time of samples detection and the Y-axis corresponds to the ions current count per second. **B**: Total ions current of mixed standard quality control samples analyzed by Agilent MassHunter. The X-axis corresponds to the retention time of samples detection and the Y-axis corresponds to the ions current count per second.
